# Strong Neck Accumulation of ^131^I Is a Predictor of Incomplete Low-Dose Radioiodine Remnant Ablation Using Recombinant Human Thyroid-Stimulating Hormone

**DOI:** 10.1097/MD.0000000000001490

**Published:** 2015-10-02

**Authors:** Keisuke Enomoto, Yoshiharu Sakata, Kazuyuki Izumi, Yukinori Takenaka, Miki Nagai, Kazuya Takeda, Yukie Enomoto, Atsuhiko Uno

**Affiliations:** From the Otolaryngology-Head and Neck Surgery, Osaka General Medical Center, Osaka, Japan.

## Abstract

The purpose of this study was to identify the factors that predict incomplete low-dose radioiodine remnant ablation (RRA) with recombinant human thyroid-stimulating hormone (rhTSH) and to report the adverse events associated with this treatment.

Between 2012 and 2014, 43 consecutive patients with thyroid cancer received low-dose RRA with rhTSH after total thyroidectomy. We retrospectively investigated the adverse events during low-dose RRA and during diagnostic whole body scan (DxWBS) using rhTSH, and analyzed the rate of RRA completion and the associations between RRA completion and various clinical/pathological factors.

Complete RRA was seen in 33 (76.7%) patients, and incomplete RRA was observed in 10 (23.3%). Patients with incomplete RRA had stronger neck accumulation of ^131^I than those with complete RRA (*P* < 0.001). Adverse events at RRA and DxWBS were seen in 12 and 9 patients, respectively. All events at RRA were grade 1, with one exception (grade 2 vertigo after rhTSH administration). The rate of adverse events at DxWBS was significantly higher in patients with adverse events seen at RRA (risk ratio, 3.778, *P* = 0.008).

Strong neck accumulation of ^131^I is significant independent predictor of incomplete low-dose RRA. The risk of adverse events at DxWBS was higher in patients who experienced adverse events at RRA than in those who did not.

## INTRODUCTION

Although high cure rates are generally achieved after the initial treatment in patients with papillary and follicular thyroid cancer,^1–4^ patients with extrathyroidal invasion, lymph node metastasis, ^18^F-fluorodeoxyglucose-positive tumors, or *BRAF* mutations have a high risk of recurrence and death.^5–9^ In such cases, radioiodine remnant ablation (RRA) is used to administer ^131^I after total thyroidectomy in an effort to destroy both the normal thyroid remnant and microscopic residual disease. The rationale for the use of RRA is to decrease the risk of clinical tumor recurrence and to improve the sensitivity and specificity of follow-up testing via periodic serum thyroglobulin (Tg) measurement and radioiodine scanning.^10–13^ Verburg et al^14^ showed that successful RRAs could reduce the recurrence rates and improve survival rates, and that no survival difference after complete RRA was observed between initially low-risk and high-risk patients.

Historically, RRA has been performed after withholding thyroid hormone (levothyroxine) replacement for 4 or more weeks to increase endogenous thyroid-stimulating hormone (TSH) production as a means to promote ^131^I radioiodine uptake and retention in the remnant thyroid cells.^15,16^ Recombinant human TSH (rhTSH) (Thyrogen; Genzyme Corp., Cambridge, MA) has been developed as a source of exogenous TSH and has been approved for RRA and diagnostic use in patients with differentiated thyroid cancer.^17^ Use of rhTSH avoids the consequences of prolonged thyroid hormone withdrawal (THW), including symptoms of hypothyroidism such as brain, heart, liver, and kidney dysfunctions, and has been shown to improve the quality of life of the patients.^18,19^ Further, Mallick et al^20^ and Schlumberger et al^21^ reported that the rates of RRA completion did not differ in patients receiving low-dose or high-dose RRA under rhTSH or THW; therefore, low-dose RRA with rhTSH is preferred to achieve a high rate of RRA completion and to avoid the adverse effects associated with THW and high levels of ^131^I.

However, the predictors of incomplete RRA have not been identified in patients receiving low-dose RRA with rhTSH. For this reason, we here aimed to identify the factors that predict incomplete low-dose RRA with rhTSH. In addition, we also report on our experience with low-dose RRA with rhTSH and the adverse events associated with the administration of rhTSH and ^131^I.

## MATERIALS AND METHODS

### Study Population

Between July 2012 and July 2014, low-dose RRA with rhTSH stimulation was performed in 50 thyroid cancer patients after total thyroidectomy, with or without neck dissection, at our medical center. The absence of distant metastasis was confirmed in all patients via chest computed tomography (CT) and bone scintigraphy or positron emission tomography/CT if necessary. Seven patients were excluded from this study: 3 patients have a history of ^131^I administration, and 1 patient each discontinued ablation because of vertigo after rhTSH administration, refusal of diagnostic whole body scans (DxWBSs) because of the distress associated with an iodine diet, cardiac infarction at 5 months after RRA, and distant metastasis developing in the lung, resulting in 43 patients being analyzed. All patients provided written informed consent to participate in this study.

### RRA With rhTSH Stimulation

**FIGURE 1:**
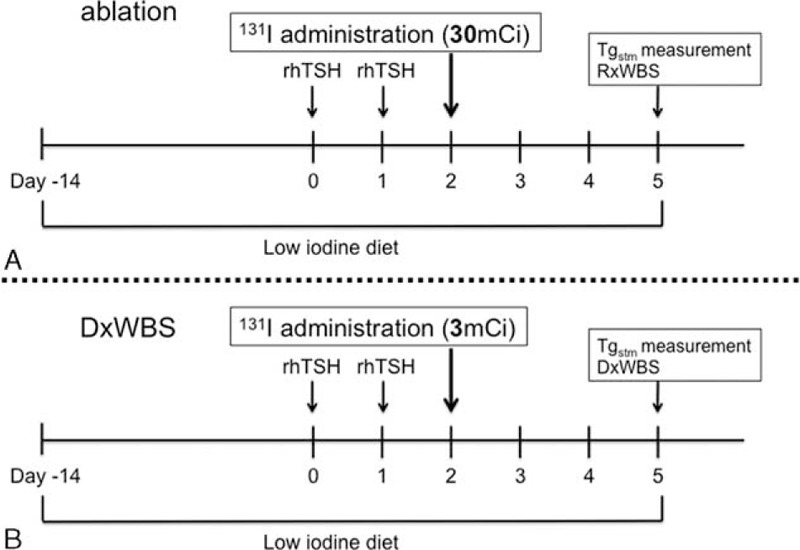
Protocol for recombinant human thyroid-stimulating hormone (rhTSH) and ^131^I administration. The dose of ^131^I used for remnant thyroid ablation was 30 mCi (A), and the dose of ^131^I used for follow-up diagnostic whole-body scanning (DxWBS) was 3 mCi (B). RxWBS = posttherapy whole body scan.

The protocol for rhTSH administration for both therapeutic and diagnostic purposes is shown in Figure 1. Briefly, after adhering to a low-iodine diet for 2 weeks, the patients underwent stimulation with rhTSH. rhTSH was administrated for 2 days (0.9 mg, intramuscularly), and the levothyroxine administration was not stopped. Serum samples for Tg measurement were collected the 14–28 days before the first administration of rhTSH and on the 4 days after the last administration of rhTSH. The day after the last injection of rhTSH, ^131^I (30 mCi; 1110 MBq) was administered for therapeutic purposes (Fig. 1A). A posttherapy whole body scan (RxWBS) was acquired 3 days after ^131^I administration. Approximately 6 months after therapy, DxWBSs and Tg measurements were performed under the same protocol of ablation in all patients; 3 mCi (111 MBq) ^131^I was administrated for DxWBS (Fig. 1B).

RxWBS and DxWBS images were obtained using a double-head gamma camera (Brightview; Philips Healthcare, Bothell, WA) with a 3/8-inch-thick crystal and a high-energy, general-purpose collimator. These images, with anterior and posterior views, were acquired after scanning at a rate of 13 cm/minute. Anterior neck spot views were acquired after scanning for a minimum of 7.5 minutes. Thyroid bed uptake was diagnosed on whole body or neck spot view images as visible uptake between the suprasternal notch and thyroid cartilage. When abnormal uptake of ^131^I was observed, a single-photon emission CT image was acquired.

### Clinical Data Acquisition

**FIGURE 2:**
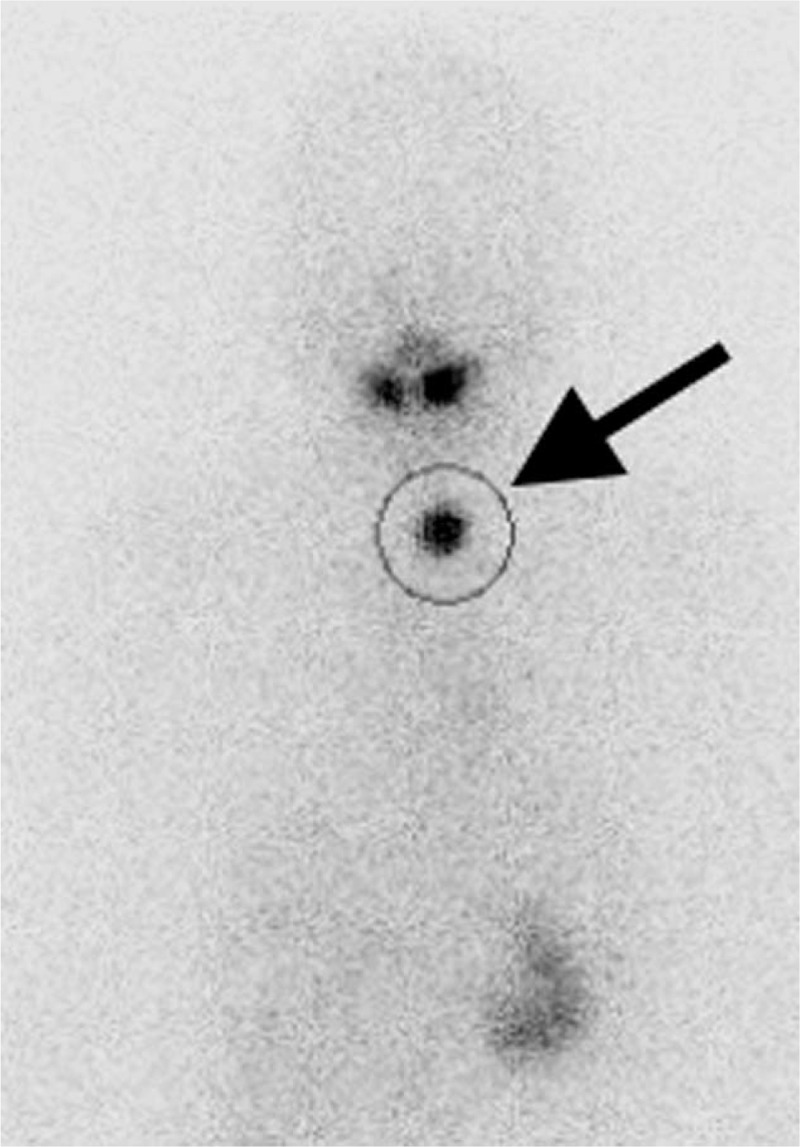
Accumulation in the neck was calculated as neck maximum count per pixel (arrow). Region of interest was established the estimated location of the thyroid.

Clinical data were obtained retrospectively. These data included age, sex, weight, pathology (papillary, follicular, or poorly differentiated carcinoma), kidney function, timing of RRA (RRA after initial surgery or surgery for recurrence), neck accumulation of ^131^I (strength and number), interval from RxWBS to DxWBS, serum Tg levels under rhTSH stimulation (Tg_stm_), serum Tg levels before rhTSH administration (Tg_pre_), Tg antibody (Tg-Ab) levels, and RRA completion (complete or incomplete). The strength of neck accumulation of ^131^I was calculated by the maximum count per pixel (Fig. 2). Body mass index (BMI) was calculated as weight (kg)/height^2^ (m), and body surface area (BSA) was calculated as weight^0.425^ (kg) × height^0.725^ (cm) × 0.007184.^22^ Adverse events during RRA and DxWBS with rhTSH were recorded according to the Common Terminology Criteria for Adverse Events (CTCAE) version 4.0. RRA completion was defined as no visible thyroid bed uptake of ^131^I on DxWBS by consensus of at least 2 specialists in nuclear medicine.

### Statistical Analysis

The χ^2^-test was used to investigate the associations between adverse events and RRA and DxWBS. Univariate analyses using the χ^2^-test or Wilcoxon/Kruskal-Wallis test were performed to assess the associations of the clinical parameters (sex, age, weight, BMI, BSA, and kidney function) with the presence of adverse events after rhTSH administration at RRA. Potential predictive variables (sex, age, weight, BMI, BSA, pathology, surgery preceding RRA, neck accumulation of ^131^I, kidney function, Tg-Ab, Tg_stm_ at ablation, Tg_stm_ at DxWBS, Tg_pre_ at ablation, Tg_pre_ at DxWBS, and interval from RxWBS to DxWBS) were compared between patients with complete and incomplete RRA. A *P* value less than 0.05 was considered statistically significant, and all analyses were performed using JMP 11.0.0 statistical software (SAS Institute, Cary, NC).

## RESULTS

The clinical data are summarized in Table 1. There were 17 (39.5%) men and 26 (60.5%) women in our study. The median age was 64.6 years (range, 27.4–81.8 years), and the median weight was 57 kg (range, 40–94 kg). The calculated BMI and BSA were 23.0 kg/m^2^ (range, 16.4–32.4 kg/m^2^) and 1.621 m^2^ (range, 1.315–2.147 m^2^), respectively. Forty-two (97.7%) patients had papillary thyroid cancer, and 1 (2.3%) patient had poorly differentiated cancer; there were no cases of follicular thyroid cancer. Kidney function was normal and abnormal in 41 (95.3%) and 2 (4.7%) patients, respectively. Thirty (69.8%) RRAs were performed after initial surgery, and 13(30.2%) RRAs were performed after surgery for recurrent disease. The median strength of neck accumulation of ^131^I on RxWBSs was 64 (range, 5–1365), and 21 (48.8%) and 22 (51.2%) patients showed multiple and single accumulation, respectively. The median interval from RxWBS to DxWBS was 196 days (range, 161–252 days). Twelve (27.9%) patients were positive for Tg-Ab. Twenty-three (53.5%) patients showed Tg_stm_ over 5 ng/mL at RRA and 20 (46.5%) patients at DxWBS. Otherwise, 6 (14.0%) patients showed Tg_pre_ over 5 ng/mL at RRA and 8 (18.6%) at DxWBS. Complete RRA was noted in 33 (76.7%) patients, while incomplete RRA was observed in 10 (23.3%).

**TABLE 1:**
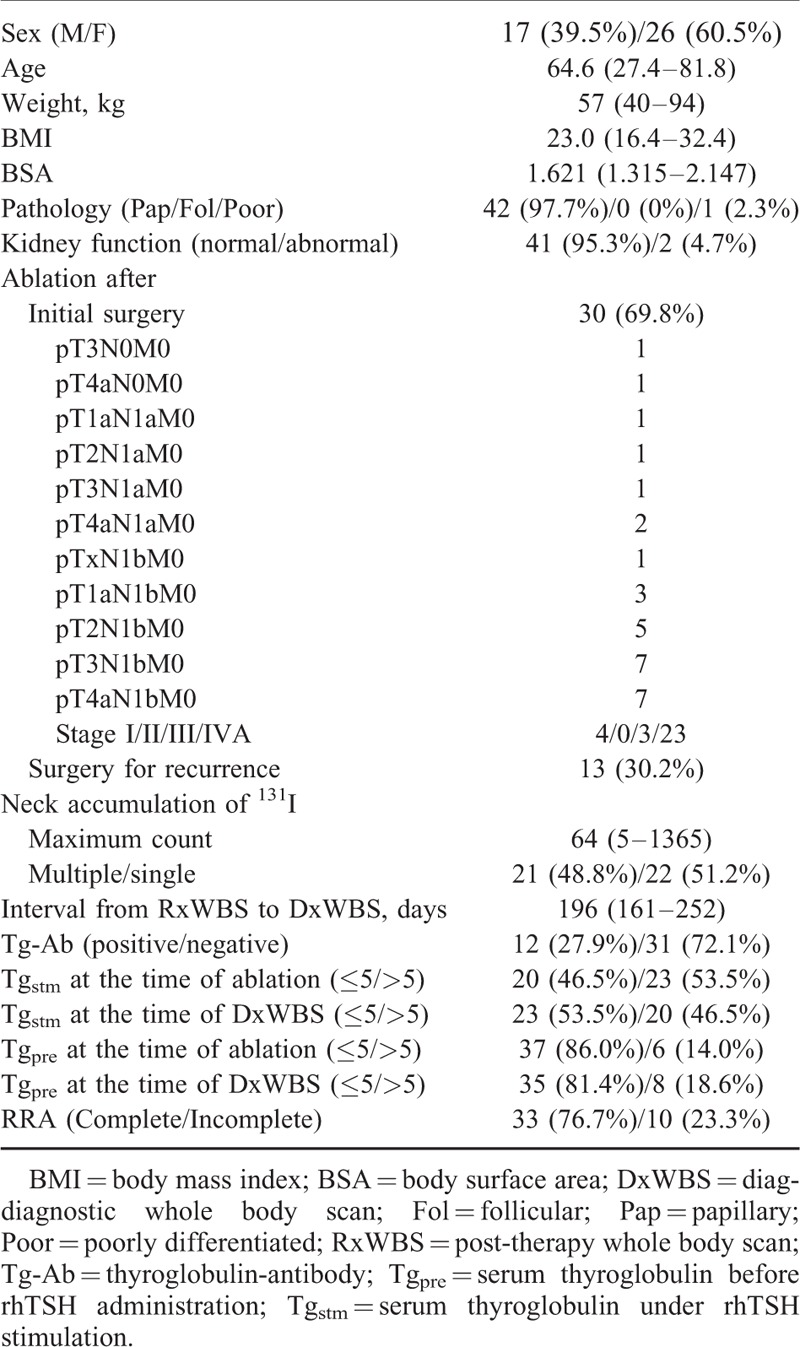
Characteristics of Patients Receiving Radioiodine Remnant Ablation (RRA) With Recombinant Thyroid-Stimulating Hormone

### Adverse Events at RRA Using rhTSH

Adverse events at RRA and DxWBS were seen in 12 (26.1%) and 9 (19.6%) patients, respectively. The different types of adverse events are listed in Table 2. All were grade 1 events with 1 exception (grade 2 vertigo after rhTSH administration). The rate of adverse events at the time of DxWBS was significantly higher in patients with the adverse events at RRA (risk ratio, 3.778; 95% confidence interval, 1.598–8.933, *P* = 0.008). The candidate clinical parameters were compared in patients with or without adverse events after administration of RRA with rhTSH (Table 3); however, no significant associations were observed.

**TABLE 2:**
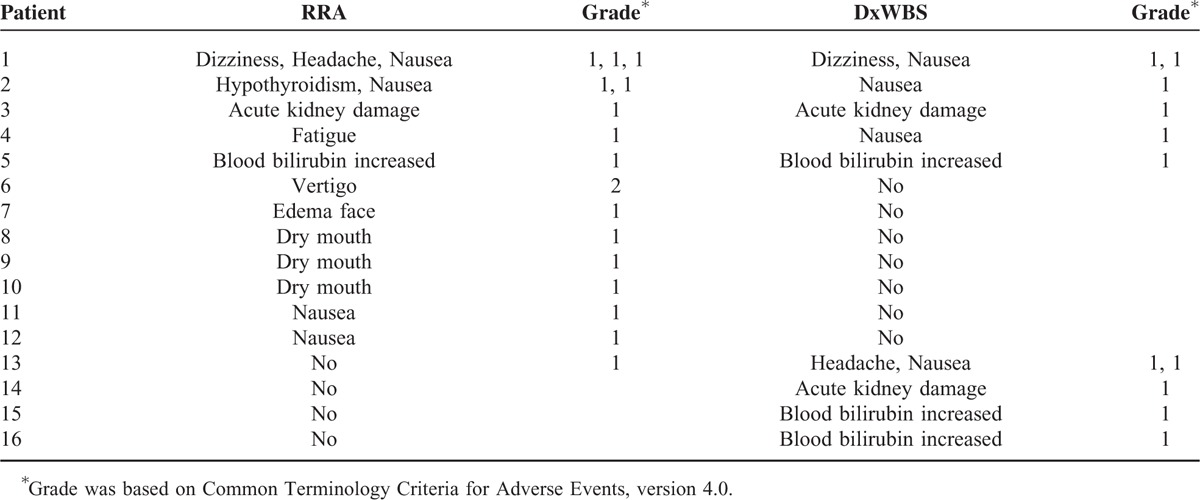
Adverse Events at Radioiodine Remnant Ablation (RRA) and Diagnostic Whole Body Scans (DxWBSs)

**TABLE 3:**
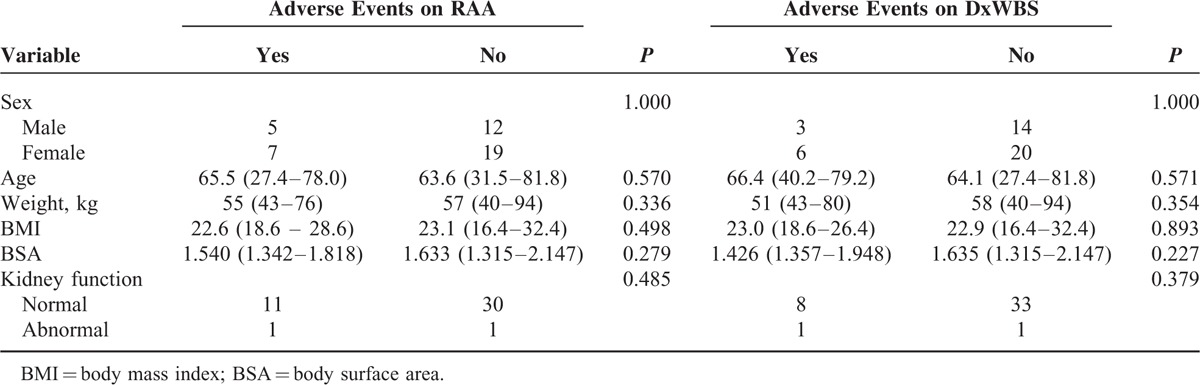
Relationship Between Adverse Events and Clinical Parameters

### Incomplete Predictors of RRA Using rhTSH

**FIGURE 3:**
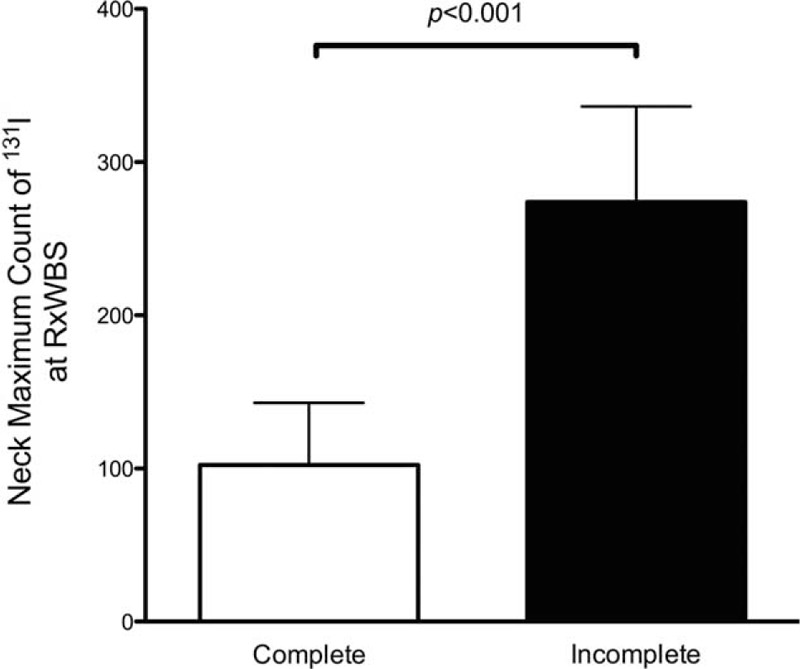
Comparison of the neck maximum count of ^131^I at RxWBS. The value in patients with RRA incomplete (n = 10) was significantly higher than that in patients with RRA complete (n = 33). Data are shown as mean ± standard error. RRA = radioiodine remnant ablation, RxWBS = post-therapy whole body scan.

Compared with patients with complete RRA, those with incomplete RRA showed stronger neck accumulation of ^131^I (*P* < 0.001, Fig. 3), as determined using univariate analysis. Heavy weight and larger BSA tend to result in incomplete RRA but not statistically significant (*P* = 0.095 and 0.070). Other clinical parameters were not different between patients with complete RRA and those with incomplete RRA (Table 4).

**TABLE 4:**
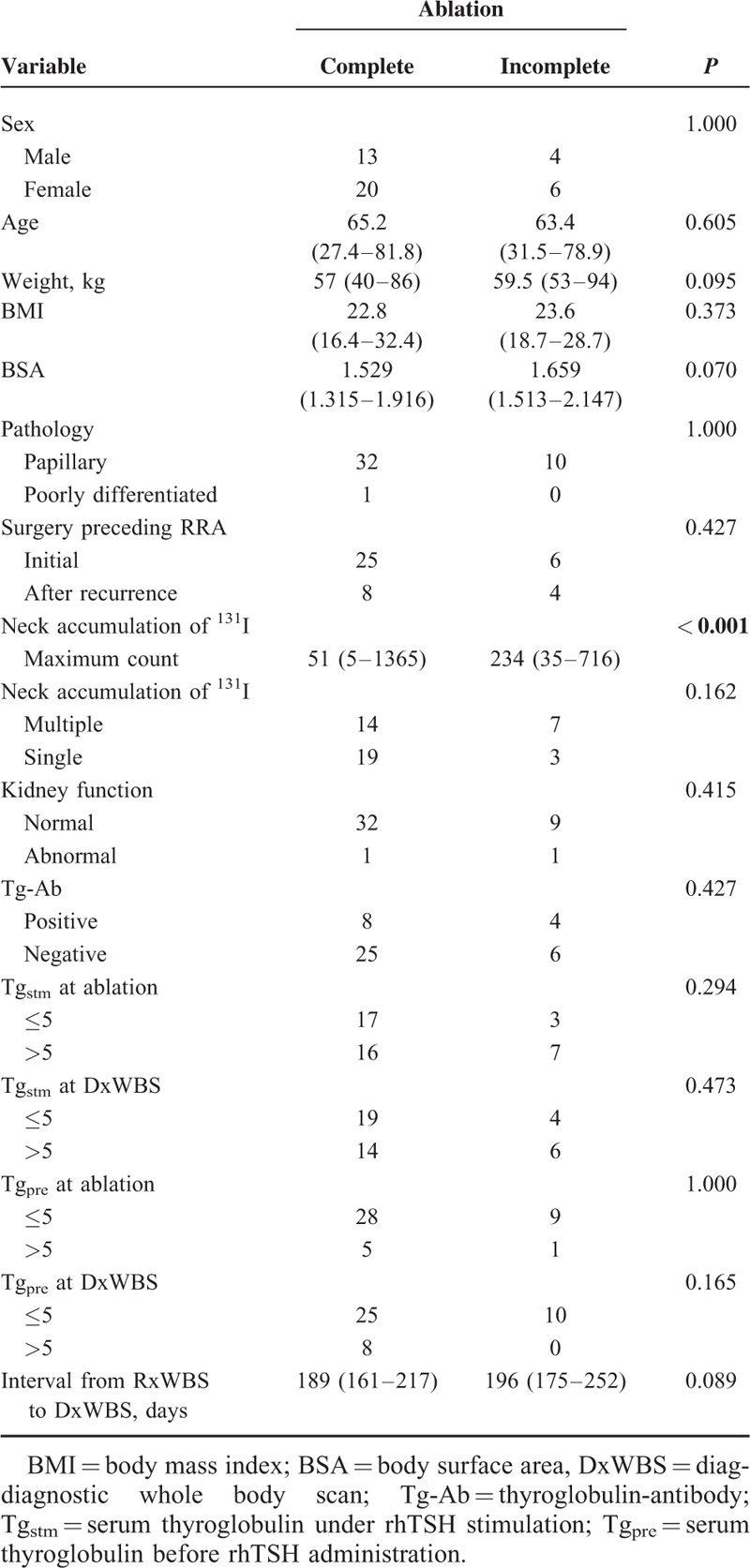
Association of Complete Radioiodine Remnant Ablation (RRA) With Clinical Parameters in a Univariate Analysis

## DISCUSSION

To date, only a few reports have assessed the potential predictors of complete RRA.^23–25^ In an analysis of 449 patients administered ^131^I (<50 mCi) after THW, Verburg et al^24^ concluded that the absorbed dose predicted complete RRA. However, because the absorbed dose is indicated by D (Gy) = dε (J)/dm (kg), where D is the absorbed dose, we speculated that the rate of complete RRA may be influenced by weight and BSA. Castagna et al^26^ previously reported that there were negative correlations between serum peak TSH levels and factors related to human body composition, such as weight (*r* = 0.39), BSA (*r* = 0.47), BMI (*r* = 0.24), and lean body mass (*r* = 0.50) in 105 patients undergoing low-dose RRA using rhTSH. They also discussed the question the rhTSH dose should be personalized arises for ablation, and expected that the serum peak TSH levels did not play a major role for complete RRA because of other parameter such as the thyroid remnant volume, the effective half-life of radioactivity taken up by residual thyroid tumor after ^131^I therapy, or the initial dose rate delivered to the tissue. In this study, the weight and the BSA tend to be heavier and larger in patients with incomplete RRA than in patients with complete RRA, but not statistically significant. Hence, the large number analysis may show that the human body composition affects the ratio of RRA completion.

Instead, our results suggested strong neck accumulation of ^131^I as an independent predictor of incomplete RRA (*P* < 0.001, Fig. 3). Previously, Logue et al^25^ reported that there was no relation between initial iodine uptake and RRA completion in their study of 121 patients. However, their study design had significant limitations. Importantly, their patients underwent various kinds of surgery, such as lobectomy without completion thyroidectomy and subtotal thyroidectomy, and the different study conditions between the studies, such as the type of surgery and use of rhTSH, may be responsible for the discrepancies in the results. Our findings indicate that when strong neck accumulation of ^131^I is noted at RRA, a subsequent RRA should be considered instead of DxWBS at 6 months after the initial RRA.

Additional operations, including surgery for recurrent foci, are associated with a high risk of surgical complications, especially recurrent laryngeal nerve palsies (RLNP), even for benign thyroids.^27,28^ Bilateral RLNP requires a tracheostomy and can cause issues with aspiration. Recurrent surgery, including complete thyroidectomy, may leave more thyroid tissue than initial surgery, including total thyroidectomy, because it is difficult for the surgeon to remove the thyroid tissue in the scar while preventing RLNP. Therefore, we also assessed the relationship between the completion of RRA and surgery for recurrence. RRA after recurrence surgery tended to be associated with a poorer completion rate than RRA after primary surgery; however, this different was not significant (*P* = 0.427).

Although, completion of RRA has been demonstrated to improve the patient prognosis, the definition of complete RRA is still controversial, and the assessment is problematic.^29^ When the level of Tg markedly increases after RRA, recurrent growth of the tumor is indicated. In the study by Schlumberger et al,^21^ 315 patients (43%) had Tg levels of <1 ng/mL without Tg-Ab production before RRA, and in a study by Mallick et al,^20^ 110 patients (25%) had subthreshold Tg levels before RRA. Our study included 20 patients (46.5%) whose serum Tg_stm_ levels at the time of RRA were ≤5 ng/mL, and the Tg-Ab was positive in 12 patients (27.9%). Additionally, not only rhTSH, but also the endogenous TSH condition, strongly affects the serum Tg_stm_ level; for this reason, we did not use the Tg level as a criterion for complete RRA in this study.

Our present study clearly showed that strong neck accumulation of ^131^I associates to incomplete RRA, but it still has several limitations. First, the clinical stage of thyroid cancer we treated was higher than those of previous reports.^20,21^ RRA for advanced thyroid cancer may lead to poor ratio of RAA completion by residual disease. Second, a multiple doctors performed the thyroidectomy in the present study, and it may associate of the thyroid remnant volume.

Finally, we also investigated the adverse events associated with low-dose RRA with rhTSH. Adverse events < grade 2 were seen in 27.9% of the patients; however, these results do not include a patient who withdrew from the study after rhTSH administration because of grade 2 vertigo, and the actual rate of adverse events is considered relatively high. The adverse events after administration of low-dose RRA with rhTSH included nausea, acute renal injury, vertigo, headache, fatigue, increased levels of blood bilirubin, dry mouth, facial edema, and dizziness. Because the adverse events were not associated with clinical parameters such as age, sex, or BSA (Table 3), it was not possible to predict the high-risk patients at the time of RRA with rhTSH. However, our results showed that the rate of adverse events at the time of DxWBS was significantly higher in patients with the adverse events at RRA (risk ratio, 3.778, *P* = 0.008). Nevertheless, the events observed in our study were almost exclusively grade 1 events, and were thus considered mild, suggesting that DxWBSs using rhTSH may be tolerable in patients with adverse reactions after low-dose RRA with rhTSH.

## CONCLUSIONS

Completion of low-dose RRA using rhTSH postsurgery was observed in 76.7% of the patients in this study. Strong neck accumulation of ^131^I was significant independent predictor of incomplete low-dose RRA with rhTSH. Adverse events occurred in 27.9% of patients and were generally mild and easily treated. Lastly, the risk of adverse events at DxWBS was significantly higher in patients who experienced adverse events at RRA than in those who did not.
